# Helminth Immunomodulation in Autoimmune Disease

**DOI:** 10.3389/fimmu.2017.00453

**Published:** 2017-04-24

**Authors:** Taylor B. Smallwood, Paul R. Giacomin, Alex Loukas, Jason P. Mulvenna, Richard J. Clark, John J. Miles

**Affiliations:** ^1^School of Biomedical Sciences, The University of Queensland, Brisbane, QLD, Australia; ^2^Centre for Biodiscovery and Molecular Development of Therapeutics, Australian Institute of Tropical Health and Medicine, James Cook University, Cairns, QLD, Australia; ^3^QIMR Berghofer Medical Research Institute, Brisbane, QLD, Australia; ^4^Division of Infection and Immunity, Cardiff University School of Medicine, Cardiff, UK; ^5^School of Medicine, The University of Queensland, Brisbane, QLD, Australia

**Keywords:** helminthic therapy, autoimmunity, immunomodulation, excretory/secretory products, immunotherapy

## Abstract

Helminths have evolved to become experts at subverting immune surveillance. Through potent and persistent immune tempering, helminths can remain undetected in human tissues for decades. Redirecting the immunomodulating “talents” of helminths to treat inflammatory human diseases is receiving intensive interest. Here, we review therapies using live parasitic worms, worm secretions, and worm-derived synthetic molecules to treat autoimmune disease. We review helminth therapy in both mouse models and clinical trials and discuss what is known on mechanisms of action. We also highlight current progress in characterizing promising new immunomodulatory molecules found in excretory/secretory products of helminths and their potential use as immunotherapies for acute and chronic inflammatory diseases.

## Introduction

Helminths are large multicellular organisms that can be either free living or parasitic. Parasitic helminths comprise the phyla of roundworms (nematodes), flatworms (platyhelminths), tapeworms (cestodes), and flukes (trematodes) and have plagued humans and archaic humans for hundreds of thousands of years. Today, these parasites remain one of the most successful families of infectious agents on the planet, infecting more than one and a half billion people (1). In humans, heavy infection with parasites can lead to many serious health problems and sometimes even death (2, 3). However, a small worm burden typically has limited or no pathology and has even been suggested to be commensal to the host (4).

## Ancient Cloakers

**Figure 1:**
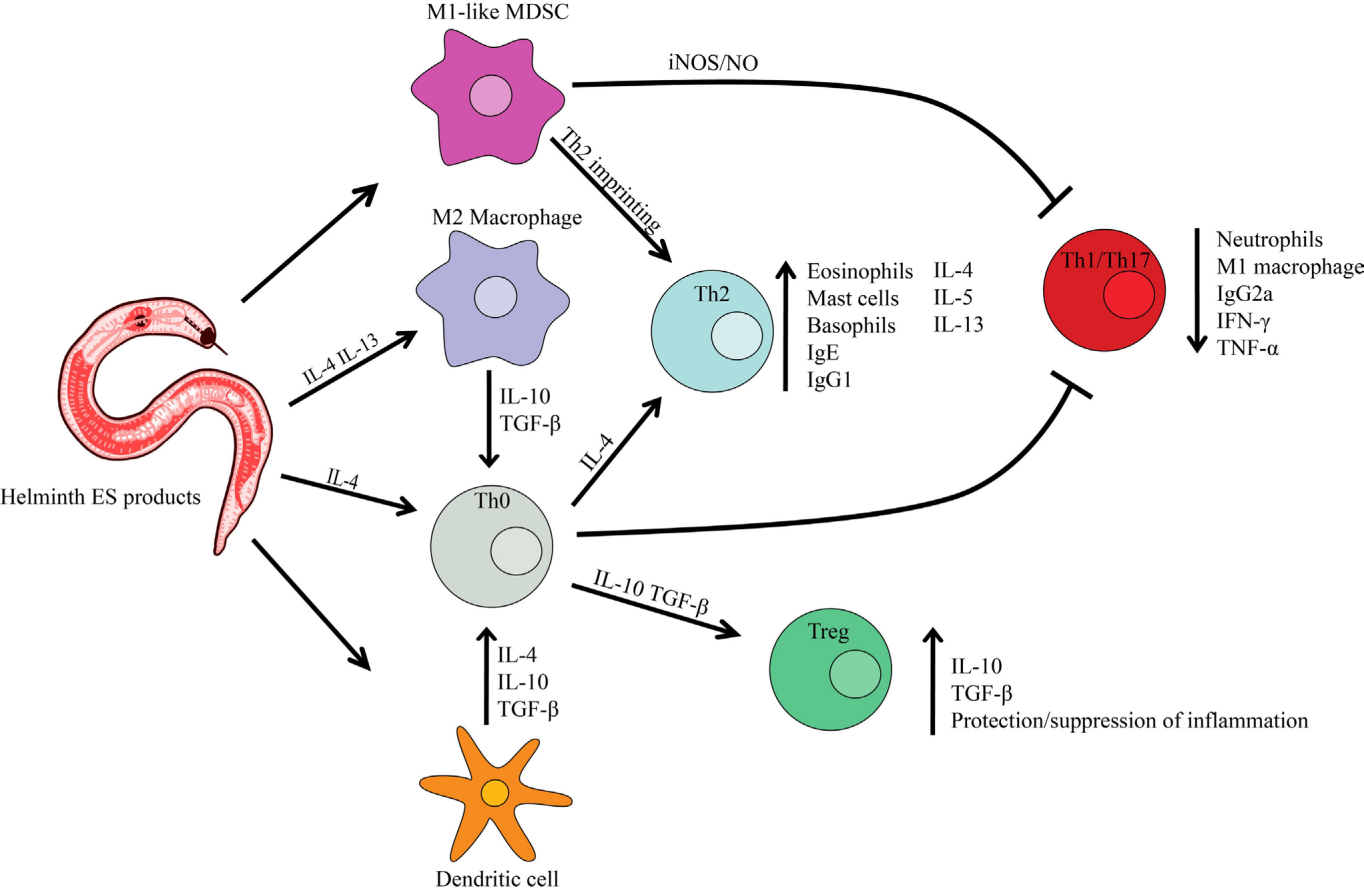
**Helminth excretory/secretory (ES) products effect on host immune cells**. Infection with parasitic worms causes the host immune system to polarize into a Th2 response (preventing Th1 or Th17 immune response) characterized by Th2 cytokines. Helminth ES products can cause the differentiation of macrophages toward the M2 phenotype, resulting in a Th2 immune response. ES products can also prevent dendritic cell synthesis of pro-inflammatory cytokines and promote the production of immunoregulatory molecules such as IL-10 and TGFβ. A regulatory T cell (Treg) phenotype is also induced, promoting the protection/suppression of inflammation produced by a Th1 autoimmune disease. Myeloid-derived suppressor cells (MDSC) function as immunoregulators, producing reactive oxygen/nitrogen species that inhibit the function of T cells.

Individual hookworms can live in the human intestine for up to 18 years (5). To achieve this impressive feat, the parasites effectively cloak through multipronged immunomodulation. The principal immune subsystem targeted is T cell surveillance (6, 7), which determines self from foreign antigens through a vast yet structured *in vivo* T cell receptor repertoire (8). Specifically, the parasites stimulate the release of IL-4, IL-5, IL-10, and IL-13, which promotes Th2 polarization (9, 10) (Figure 1). Regulatory T cell (Treg) development is also stimulated during hookworm infection (11) that enhances the cloaking effect through the release of the regulatory cytokines IL-10 and transforming growth factor (TGF) β (12). In addition, hookworms induce activation of parasite-specific and total immunoglobulin E (IgE) and the mobilization of the innate immune systems including mast cells, eosinophils, and basophils (13). Indeed, a recent large-scale community deworming study showed that helminths actively decrease immune responsiveness and modulate immune checkpoint expression in infected individuals (14). The intrinsic talent of parasitic worms to skew the immune response from Th1 to Th2/Treg has led to the idea of using live worms as immunotherapy (helminthic therapy) or, preferably, seeking compounds in helminth secretions for use as immunomodulatory drugs. Indeed, helminthic therapy in animal models and human trials has provided convincing evidence that low-dose inoculation can treat a number of autoimmune diseases.

## Increasing Burden of Autoimmune Disease

Autoimmunity is the failure of the immune system to distinguish pathogens from self-antigens resulting in damage to healthy tissue (15). Today more than 80 autoimmune diseases have been identified, including inflammatory bowel disease (IBD), multiple sclerosis (MS), rheumatoid arthritis (RA), and type 1 diabetes (T1D) (16). Autoimmune diseases are now estimated to affect almost 10% of the world’s population and collectively represent truly massive global disease and financial burdens (17). Most autoimmune diseases have no cures and are not knowingly preventable. Disconcertingly, for several decades, the developed world has seen steady increasing incidence of autoimmune disease (18–21). While genetic predisposition is known to be a key factor in susceptibility (22), the sudden surge in these diseases over a very short time period cannot be explained by genetics alone, but rather points to variations in environment and/or lifestyle (23, 24). Two major theories have been put forward to explain this epidemiology including the “hygiene hypothesis” and the “old friends’ hypothesis” (25, 26).

## Dirty Old Friends

The hygiene hypothesis, formulated in 1989, proposed that lower intensities of infections during early childhood could explain the emergence of asthma and hay fever later in life (25). The study suggested that declining family size, improvements in household amenities, and increases in personal cleanliness reduced opportunities for cross infections in young families, resulting in a more widespread clinical expression of atopic diseases. Over time, this theory has broadened to include a catalog of chronic inflammatory diseases. Indeed, urban migration, increased access to clean water, and improved sanitation have reduced exposure to many infectious agents including helminths (27). Multiple epidemiological studies have shown an inverse correlation between microorganism exposure and the development of autoimmunity (28–33).

Concordantly, the old friends’ hypothesis suggests that various organisms, including helminths and microbiotas, have long coevolved with their mammalian hosts and act as inducers of immunoregulatory circuits (24, 34). This hypothesis has a sound rationale given that infectious agents, including helminths, are known to be potent modulators of T cell function and that dysregulation of T cell subsets (Th1 and Th17) are fundamental in autoimmune disease processes (35–37) including MS (38), RA (39), and psoriasis (40). Of note, an inverse association has been observed between the prevalence of certain helminths and autoimmune diseases (24).

## Animal Models of Helminth Therapy

Over the last decades, there have been numerous animal models used to study hookworm therapy for autoimmune disease (IBD, MS, RA, and T1D). Although these individual animal models do not fully reflect the pathology of human disease, the data obtained can be used for safety and at the very least predictive for therapeutic efficacy in humans. The following sections detail current animal models of helminth therapy and therapy with helminth-derived secretory products.

### Inflammatory Bowel Disease

Inflammatory bowel disease is characterized by a chronic relapsing inflammatory condition of the gastrointestinal tract. IBD primarily encompasses ulcerative colitis (UC) and Crohn’s disease (CD) (41). IBD pathogenesis is thought to involve dysregulation in mucosal immunity (42) and defects at the mucosal barrier, particularly a “leaky” intestinal epithelial barrier with impaired tight-junction formation can cause mucosal inflammation secondary to luminal antigen uptake (43, 44). While both diseases are forms of IBD, the autoimmune T cell responses exhibit different biology (45). CD is driven by a Th1/Th17 response with large amounts of IFNγ, IL-12, and IL-23 playing key roles. In contrast, UC is considered a Th2-mediated disease, where increases in IL-5 and IL-13 drive pathology through chronic inflammation (45).

Similar to CD, mouse models of experimental colitis trigger a Th1 type immune response, reflected by the infiltration of IFNγ-producing T cells in the colon (46). There are three types of animal models of IBD. These are broadly divided into (i) chemically induced models; (ii) models with experimentally altered immune responses; and (iii) models with intestinal epithelial defects (47). Chemically induced colitis models including the trinitrobenzene sulfonic acid (TNBS) model, dinitrobenzene sulfonic acid (DNBS) model, and dextran sodium sulfate (DSS) model are the most common platforms for IBD research. In the TNBS and DNBS models, colitis is induced *via* intrarectal instillation of the chemicals. In the DSS model, colitis is induced orally. Each model triggers a Th1 pro-inflammatory immune response within the intestine (48). A second broad model for IBD includes varieties of knockout mice (TGFβ1^−/−^, IL-10^−/−^, and STAT3^−/−^) that aid in the study of innate and adaptive immune responses during disease (49). These strains also allow for mechanistic investigations during acute or chronic enteritis. For instance; IL-10^−/−^ mice develop spontaneous colitis that is characterized by histological findings similar to those of human IBD (50). The T cell transfer model has become one of the most widely used models to study pancolitis and chronic transmural inflammation in the intestine (49, 51). This method involves the adoptive transfer of naïve T cells (CD4^+^CD25^−^) into immunocompromised mice (52). Advantages of this method include early investigation of immunological events associated with the induction of gut inflammation and the ability to study the role of Tregs in inflammation. The final type of animal model of IBD is defective intestinal epithelial responses (53). Mouse models such as IKK-γ (NEMO), IKK-β, and mdr1a^−/−^ develop spontaneous colitis due to compromised immunity at the epithelial cell wall. Many of these animal models of IBD show that colitis can be attenuated with prior exposure to different helminth species (54–59) (Table 1). Several of the parasites use the same immune regulatory mechanism, such as a Th2 polarization, which suppresses inflammation. These effects are commonly mediated through increases of cytokines including IL-4, IL-10, and IL-13 production, as well as a decrease in the pro-inflammatory cytokines such as IFNγ and TNFα (Table 1).

**Table 1:** **Helminth therapy in animal models of human autoimmune diseases**.

Animal model	Helminth species	Outcomes	Reference
**Inflammatory bowel disease**			
Trinitrobenzene sulfonic acid (TNBS)	*Schistosoma mansoni*	Helminth infection attenuates TNBS-induced colitis *via* Th2 polarization. Mediated through increases in IL-4 and IL-10 and decreases in IFNγ	(54, 55)
TNBS	*Heligmosomoides polygyrus*	Helminth infection attenuates TNBS-induced colonic injury and inflammation *via* Th2 polarization. Mediated through increases in IL-4 and IL-13	(60)
TNBS	*S. cercariae*	Both infection with helminth and immunization with recombinant P28GST attenuates TNBS-induced colitis. Mediated through Th2 polarization and modulation of eosinophil recruitment	(61)
TNBS	*Schistosoma japonicum*	Ova infection prevents TNBS-induced colitis *via* Th2 polarization. Mediated through increases in IL-4, IL-5, and IL-10 and decreases in IFNγ	(56, 62, 63)
Dextran sodium sulfate (DSS)	*S. mansoni*	Helminth infection attenuates DSS-induced colitis. Egg injections are ineffective. Mediated through macrophage trafficking	(64)
DSS	*Anisakis simplex*	Therapeutic treatment with recombinant rAs-migration inhibitory factor protein attenuates DSS-induced colitis. Thought to be mediated through regulatory T cell (Treg) expansion and increases in IL-10	(65)
DSS	*Acanthocheilonema viteae*	Therapeutic treatment with recombinant cystatin protein attenuates DSS-induced colitis. Thought to be mediated *via* targeting and modulation of macrophages	(66)
Dinitrobenzene sulfonic acid (DNBS)	*Trichinella spiralis*	Helminth infection reduced severity of DNBS-induced colonic damage. Mediated through increases in IL-4 and IL-13 and a decrease in IFNγ	(42)
DNBS	*Hymenolepis diminuta*	Helminth infection in WT and IL-22^−/−^ mice attenuates DNBS-induced colitis. An increase in the number of mucus-containing goblet cells in the small intestine was observed in WT but not IL-22^−/−^ mice	(67)
NSAID	*Trichuris muris*	Helminth infection in Nod2^−/−^ mice restored SI goblet cell numbers/morphology and decreased IFNγ-secreting CD8^+^ T cells in the intestine	(68)
TCT	*H. polygyrus*	Helminth infection in Rag mice attenuates TCT-induced colitis. Mediated through decreases in IL-12 and IFNγ and increases in IL-13 and Treg	(69)
TCT	*H. polygyrus*	Helminth infection in Rag mice attenuates TCT-induced colitis. Mediated through altered dendritic cell (DC) function in the mucosa	(57)

**Multiple sclerosis**			
Experimental autoimmune encephalomyelitis (EAE)	*S. mansoni*	Helminth infection attenuated the clinical course of EAE. Therapeutic exposure significantly delayed the development of symptoms. Mediated through an increase of IL-4 and decrease of pro-inflammatory cytokines	(70, 71)
EAE	*T. spiralis*	Helminth infection maintained Th2 immunity after EAE induction. Transfer of T cells from infected mice to EAE immunized mice amelioration disease and protected from disease	(72)
EAE	*Fasciola hepatica*	Helminth infection attenuated the clinical course of EAE. Mediated through migration interference of DCs, macrophages eosinophils, neutrophils and CD4^+^ T cells	(73)
EAE	*S. japonicum*	Helminth infection reduced inflammation and demyelination in spinal cords. Mediated through a Th2-biased microenvironment of low IFNγ and high IL-4 production in the spleen and CNS	(74)

**Type 1 diabetes**			
Non-obese diabetic (NOD)	*S. mansoni*	Helminth infection or ova injection prevented disease if administered before the onset of pancreatic infiltration (<4 weeks of age). Mediated through a Th2-biased environment of increased IL-4, IL-5, IL-10, and IL-13	(75, 76)
NOD	*H. polygyrus*	Helminth infection protects animals from disease for <35 weeks. Thought to be mediated through Th2 skewing and modulation of IL-4 and IL-13 expression. Mechanism independent of IL-10 and CD4^+^/CD25^+^ T cells	(77, 78)
NOD	*T. spiralis*	Helminth infection protected animals from disease for <37 weeks. Thought to be mediated by increases in CD4^+^ cells and decreases in CD8^+^ and NK cells in the pancreas. Th2 skewing noted	(77)
Diabetic retinopathy	*Ancylostoma caninum*	Transgenic mice expressing neutrophil inhibitory factor (NIF) are protected from diabetic retinopathy. NIF did not compromise normal immune surveillance but did result in large amounts of superoxide	(79)

**Rheumatoid arthritis**			
CIA	*S. mansoni*	Helminth infection attenuates disease. Mediated through decreases in IFNγ, TNFα, and IL-17 and increases in IL-4 and IL-10	(80)
CIA	*S. japonicum*	Helminth infection attenuates disease incidence and severity. Protection was infection stage dependent. Mediated through decreases in IFNγ and autoantibodies and increases in IL-4 and IL-10	(81)
CIA	*A. viteae*	Prophylactic and therapeutic admiration of an excretory/secretory (ES)-62 analog attenuates disease. Mediated through decrease in inflammasome activity and IL-1β at disease site	(82)
MRL/Lpr	*H. polygyrus*	Helminth infection attenuates incidence and severity of spontaneous disease. Mediated through increases in IL-4 and IgG1 and decreases in lymphocyte infiltration at disease site	(83)

**Systemic lupus erythematosus**			
MRL/Lpr	*A. viteae*	Therapeutic administration of ES-62 analogs attenuates incidence and severity of disease. Mediated by reducing MyD88 and IL-6 in kidney infiltrating macrophages	(84)

### Multiple Sclerosis

Characterized by neurodegeneration, MS leads to the severe impairment of mobility, vision, and coordination eventually resulting in paralysis (85). The primary cause of pathology is a misdirected immune response against the myelin sheath. Damage is mediated by immunoglobulin, complement, and T cell immunity (86). Experimental autoimmune encephalomyelitis (EAE) is a mouse model of MS characterized by a pro-inflammatory T cell-mediated disease induced by priming with myelin proteins/peptides (87). CNS autoimmunity in both EAE and MS is mediated by Th1 and Th17 cells (88). Induction is thought to be dependent on the Th1 cytokine IL-12, playing a central role in macrophage activation and nitric oxide production (89). Granulocyte-macrophage colony-stimulating factor (GM-CSF) and IL-1 are also considered key cytokines involved in the pathogenesis of EAE. GM-CSF is a key cytokine produced by T cells required for susceptibility to EAE (90). IL-1β/IL-1R signaling in endothelial cells and leukocytes is critical for EAE development (91) and stimulates GM-CSF production. Together the cytokines interact to create a cycle of neuroinflammation in the CNS. Th2 cytokines appear to be protective, suggesting that Th skewing can prevent diseases or decrease disease severity. Akin to IBD, helminthic therapy in the EAE mouse model decreases the progression of EAE through the suppression of Th1 and Th17 cells and induction of Th2 cells, Tregs, and regulatory macrophages (Table 1).

### Type 1 Diabetes

Type 1 diabetes is characterized by a progressive cellular infiltration of the pancreas resulting in the destruction of insulin-producing cells (92). The non-obese diabetic (NOD) mouse provides a model of human disease through mimicking polyuria, glycosuria, weight loss, and lymphocytic infiltration of the islets of Langerhans (75, 93, 94). At 5 weeks of age, immune infiltration of the pancreas begins, ultimately ending in lymphocyte-directed destruction of β-cells (95). Pathology is dependent on CD4^+^ and CD8^+^ T cells, with the CD4^+^ population having a Th1 phenotype (96). Antigen-presenting cells including B cells, dendritic cells (DCs), and macrophages are key mediators of disease through the presentation of self-antigens. Similar to the IBD and EAE models discussed above, helminthic therapy in the NOD mouse also triggers Th2 skewing due to increases in IL-4 and IL-13 expression, ameliorating Th1-mediated disease (Table 1).

### Rheumatoid Arthritis

Rheumatoid arthritis is characterized by chronic inflammation in the joints and overexpression of the cytokines TNFα, IL-1, and IL-6 (97). Pathogenesis involves both genetic predisposition and environmental trigger(s). A number of induced and spontaneous mouse models have been developed that recapitulate features of human disease (98). Both induced and spontaneous models of RA have been shown to benefit from helminthic therapy through decreasing inflammasome activity at the site of disease and the production of Th1 cytokines such as TNFα, while increasing IL-4 and IgG1 production (Table 1).

## Clinical Trials of Helminthic Therapy in Autoimmune Disease

### Inflammatory Bowel Disease

Ten clinical trials indicate that controlled, low-dose helminthic therapy is safe in IBD and related GIT diseases, with some trials showing statistically significant efficacy at endpoint (Table 2). In 2003, an open-label phase 1 trial examined safety by exposing CD and UC patients to pig whipworm ova (99). Four patients with active CD and three patients with UC were given a single oral dose of live eggs. Patients were routinely monitored using multiple disease and quality of life indexes over a period of 12 weeks. The trial found that all patients improved clinically without any adverse events. While patients improved for a mean duration of approximately 8 weeks, three patients experienced remission relapse 12 weeks after single helminthic therapy. The study suggested that multiple doses may be required to prolong the benefit of treatment. The study also found that there were no significant clinical complications when patients received multiple doses of live eggs at 3-week intervals for 30 weeks. The group followed up with a placebo-controlled trial of 54 UC patients. The pig whipworm arm received an oral dose of live ova at 3-week intervals for 12 weeks (100). Again, whipworm therapy produced no adverse events. Between the treatment and placebo groups, statistically significant efficacy was observed at 12 weeks in two separate indices in *post hoc* analysis. One limitation of pig whipworm therapy is that humans are not the natural host and repeated dosing is required to maintain ongoing infection. In addition, given the larvae are invasive, site of infection is unpredictable with potential migration into the lymphatics and/or small blood vessels (101). The problems of repeated inoculation and unpredictable migration motivated an alternative modality. In 2006, a proof-of-concept study explored human hookworm for the treatment of CD (102). While both hookworm and whipworm possess parasite lifecycles that require development in the external environment and therefore unable to proliferate directly in the host; the hookworm is adapted to survive in humans and establish a chronic infection that can last for years from a single inoculation. This makes human hookworm an attractive therapeutic, as a defined dose can be controlled and eliminated *via* anthelmintic therapy (103). CD patients with longstanding but mostly inactive disease were inoculated with 25 or 50 live hookworm larvae in an initial and reinoculation trial. Disease index for CD patients was unchanged until week 17. After 20 weeks, clinical scores improved and five patients were in remission at week 45.

**Table 2:** **Clinical trials using helminth therapy for the treatment of autoimmune diseases**.

Trial/phase	Species	Treatment	Status	Results	Reference
**Celiac disease**					
NCT01661933 Phase 1/2	*Necator americanus*	Larvae inoculation at weeks 0 (*n* = 10) and 4 (*n* = 10), followed by small, incremental gluten challenge in 12 subjects	Complete	No serious adverse events. Ten subjects successfully completed low-dose gluten challenge	(104)
NCT00671138 Phase 2	*N. americanus*	Larvae inoculation at weeks 0 (*n* = 10) and 12 (*n* = 5) and placebo (*n* = 10). Twenty subjects challenged at 20 weeks with 16 g gluten orally per day for 5 days	Complete	Transient enteritis in five subjects. Hookworm-infected mucosa retained healthy appearance. Infection resulted in no obvious benefit on pathology	(105)
NCT00671138	*N. americanus*	Larvae inoculation at weeks 0 (*n* = 7) and 12 (*n* = 7). Seven subjects challenged at 20 weeks with 16 g gluten orally per day for 5 days	Complete	No serious adverse events. Duodenal biopsies cultured with gluten antigen produced more IL-10 and IL-5 postinfection	(106)
NCT02754609 Phase 1	*N. americanus*	Larvae inoculation at weeks 0 and 8 (*n* = 40). Placebo group included (*n* = 10)	Active		

**Ulcerative colitis (UC)**					
Phase 2	*Trichuris suis*	Oral inoculation (2,500 ova) at 2-week intervals for 12 weeks (*n* = 30). Placebo group included (*n* = 24)	Complete	Treatment cohort saw 43% improvement in disease index. No serious adverse events	(100)
NCT01433471 Phase 1	*T. suis*	Two arms. First arm, oral inoculation (2,500 ova) at 2-week intervals for 12 weeks followed by placebo for 12 weeks. Second arm, placebo for 12 weeks followed by oral inoculation (2,500 ova) at 2-weeks intervals for 12 weeks	Complete	No study results posted	

**Crohn’s disease**					
Phase 1	*T. suis*	Oral inoculation (2,500 ova) monitored over 12 weeks in 7 patients (4× Crohn’s disease, 3× UC)	Complete	Clinical improvements observed with no serious adverse events. Three patients experienced remission relapse 12 weeks after the initial dose	(99)
Phase 1	*N. americanus*	Larvae inoculation at week 0 (*n* = 9). Reinoculation between weeks 27–30 (*n* = 5)	Complete	No serious adverse events. Five patients from first inoculation were in remission at week 45	(102)
NCT01434693 Phase 1	*T. suis*	Sequential dose escalation (500, 2,500, and 7,500 ova) given orally (*n* = 27). Placebo group included (*n* = 9)	Complete	Minor adverse events seen in both placebo and treatment groups. Infection resulted in no obvious benefit to pathology. Seven thousand five hundred ova dose was safe and well tolerated	(107)
NCT01576471 Phase 2	*T. suis*	Oral inoculation (7,500 ova) at 2-week intervals for 10 weeks. Placebo group included	Unknown	Study results unknown	
NCT01279577 Phase 2	*T. suis*	Oral inoculation (low, medium, and high-dose ova) with placebo group included	Complete	Study results unknown	
NCT02281916 Phase 2	*Schistosoma mansoni*	Injections of P28GST protein (100 μg) at 1-month intervals for 3 months (*n* = 24)	Active		

**Multiple sclerosis**					
Clinical monitoring	Multiple species	Prospective clinical monitoring study of parasite-infected patients (*n* = 12) and non-infected patients (*n* = 12)	Complete	Parasite-infected patients presented with fewer numbers of exacerbations. A significant increase in IL-10 and TGFβ and a decrease in IL-12 and IFNγ observed in self-reactive cells	(108)
Clinical monitoring	Multiple species	Prospective clinical monitoring study of parasite-infected patients with relapsing-remitting disease (*n* = 12). Four patients received antiparasitic treatment over the monitoring period	Complete	After antiparasitic treatment, patients presented with increased numbers of exacerbations. This was met with a decrease in IL-10- and TGFβ-secreting cells	(109)
NCT00645749 Phase 1	*T. suis*	Oral inoculation (2,500 ova) at 2-week intervals for 12 weeks (*n* = 5). Baseline versus treatment exploratory trial	Complete	No serious adverse events. Increases in serum IL-4 and IL-10 during treatment. A trend decrease in disease index during treatment	(110)
NCT00645749 Phase 2	*T. suis*	Oral inoculation (2,500 ova) at 2-week intervals (*n* = 18)	Active, not recruiting		
NCT01006941 Phase 2	*T. suis*	Oral inoculation (2,500 ova) at 2-week intervals for 12 weeks (*n* = 10)	Complete	Well tolerated with only mild and self-limiting adverse events. Infection resulted in no obvious benefit to pathology	(111)
NCT01470521 Phase 2	*N. americanus*	Single dermal inoculation (25 larvae) at week 0 (*n* = 36). Placebo group included	Complete	Study results unknown	
NCT01413243 Phase 2	*T. suis*	Oral inoculation (2,500 ova) every 2 weeks for 12 months. Placebo group included. Total study (*n* = 50)	Terminated	Unknown	
NCT00630383 Phase 2	*N. americanus*	Single dermal inoculation (25 larvae) at week 0. Placebo group included	Withdrawn prior to enrollment	Superceded by similar study	

**Psoriasis**					
NCT01836939 Phase 1	*T. suis*	Two arms. First arm, oral inoculation (2,500 ova) every 2 weeks for 10 weeks. Second arm, oral inoculation (7,500 ova) every 2 weeks for 10 weeks. Total study (*n* = 8)	Complete	Study results unknown	
NCT01948271 Phase 1	*T. suis*	Oral inoculation (7,500 ova) every 2 weeks for 14 weeks	Terminated	Lack of efficacy	
NCT02011269 Phase 2	*T. suis*	Three arms. First arm, oral inoculation (7,500 ova) every 2 weeks for 10 weeks. Second arm, oral inoculation (15,000 ova) every 2 weeks for 10 weeks. Third arm, placebo comparator	Withdrawn	Unknown	

**Rheumatoid arthritis**					
EUCTR2011-006344-71-DE Phase 1	*T. suis*	Oral inoculation (2,500 ova) every 2 weeks for 24 weeks. Placebo group included. Total study (*n* = 50)	Prematurely ended	Study results unknown	

*Adapted and updated from Ref. (112). Information of clinical trials has been gathered from http://ClinicalTrials.gov and EU Clinical Trials registry available at the time of publication*.

Two recent human hookworm clinical trials explored the safety and efficacy of hookworm therapy in celiac disease (104, 105). The first double-blind, placebo-controlled study inoculated patients twice with 15 live hookworm larvae followed by an aggressive oral gluten challenge after patient intestinal infection was established (105). Experimental infection proved to be safe but did not result in clinical benefit following gluten challenge. Interestingly, follow-up immunological analysis found that hookworm infection altered cellular immunity (106), through decreasing basal levels of IFNγ and IL-17 in the intestine and altering CD4^+^ T cell immunity both in the intestine and, interestingly the circulatory system. The second study combined live hookworm larvae inoculation (20 larvae per individual) with desensitization, specifically a sustained gluten microchallenge (104). Of note, no uninfected controls were used in the study. Escalating gluten challenges were well tolerated and resulted in stabilization or improvement across all tested indices of gluten toxicity. IFNγ-producing intestinal T cells were observed to decrease, while Treg numbers in the epithelium increased significantly. Three human clinical trials for IBD that have been completed are yet to post study results (NCT01433471, NCT01576471, and NCT01279577) (Table 2). A larger phase 1b dose-ranging hookworm trial for celiac disease treatment is underway (NCT02754609) (Table 2).

### Multiple Sclerosis

Six clinical trials in MS have been completed or are in progress for helminthic therapy (Table 2). In 2007, a prospective study of MS patients who were recently positive for parasitic infections (and negative for the 2 previous years) were followed over approximately 5 years *via* disease score and immunomonitoring (108). The study found significantly lower disease scores and lower numbers of disease exacerbations in helminth-infected patients. Compared with uninfected patients, myelin basic protein-specific T cells in the peripheral blood showed increased IL-10 and TGFβ production and decreased IL-12 and IFNγ production. Increased success of *in vitro* cloning efficacy of Tregs was also noted in infected MS patients when compared with uninfected patients. A succeeding study followed the same relapsing–remitting MS patients with natural parasitic infections from the previous study for approximately 7 years (109). During the course of study, four MS patients received anthelmintic treatment due to worsening symptoms associated with infection. Posttreatment, there was a significant increase in disease score in these individuals accompanied by a permanent alteration of immune phenotype in the circulatory system (decreases in IFNγ-secreting cells and absolute Treg numbers). Asymptomatic, persistently infected patients maintained a significantly lower disease score across the monitoring period. It was speculated that helminths induce regulatory networks that could explain environment-related epidemiology of disease.

The first helminthic therapy trial for MS was published in 2011 (110). Here, five MS patients were given repeated oral doses of pig whipworm for 12 weeks in a baseline versus treatment-controlled exploratory trial. Results revealed that helminthic therapy was well tolerated, and some favorable trends were observed in disease scoring. Increases in serum IL-4 and IL-10 levels were noted in four of the five patients. The second helminthic therapy trial for MS was published in 2015 (111). Here, 10 MS patients were given repeated oral doses of pig whipworm for 12 weeks. Treatment was well tolerated with only mild and self-limiting adverse events. However, no positive effect on disease activity was observed, and there was no alteration in the examined immune biomarkers in the peripheral blood. For both pig whipworm trials, it is currently unknown if the relatively short infection period of 12 weeks is sufficient time to initiate clinical efficacy. Several phase 1/2 clinical trials using pig whipworm or hookworm are currently recruiting or ongoing (Table 2). In addition to IBD and MS, two helminthic therapy trials have been conducted for the treatment of other autoimmune disease such as psoriasis and RA (Table 2). However, a number of trails for MS (NCT01413243 and NCT00630383), psoriasis (NCT01948271 and NCT02011269), and RA (EUCTR2011-006344-71-DE) have been terminated or withdrawn prior to enrollment due to supersession by another study, possessing a lack of efficacy or an unknown cause.

Helminthic therapy is not without controversy. Direct treatment with living worms could cause pathology. Furthermore, the idea of being infected with a living parasite may be a difficult task for many patients. With these limitations in mind, immunomodulatory proteins and peptides secreted by helminths have become a more attractive target for drug development. Here, the use of immunomodulatory drugs derived from helminth molecule “blueprints” would provide a safer and more controllable therapeutic modality.

## Immune Modulating Excretory Secretory Products

Excretory/secretory (ES) products are the primary interface between parasitic worms and their hosts (113). ES products contain a mixture of proteins, glycoproteins, and small molecular weight compounds that are secreted from the oral openings or outer body surfaces (114). ES products are essential for helminth survival/propagation, allowing the parasites to evade immune surveillance. While a number of studies have reported the benefits of ES products in treating autoimmune diseases in mouse models, to date, only a few worm-derived immunomodulatory macromolecules and recombinant proteins have been characterized in depth. Likewise, ES proteins investigated to date represent only an infinitely small slice of the bioactive compounds found in the complex fluids of helminths. There have been multiple inventories of ES proteins generated from different types of parasitic worms including *Fasciola hepatica* (115), *Trichinella spiralis* (116), *Haemonchus contortus* (117), *Brugia malayi* (118), *Teladorsagia circumcincta* (119), *Schistosoma mansoni* (120), and *Ancylostoma caninum* (114). Many studies focus on higher molecular weight proteins (>5 kDa) (114), and there is a notable absence of research on lower MW products (1–5 kDa). Large-scale sequencing projects have revealed the presence of peptides within the genome/transcriptome of *Necator americanus* and *Ancylostoma ceylanicum* (121–123). In particular, a group of peptides highly expressed in hookworm species exhibit sequence/structural homology to the *Stichodactyla helianthus* toxin (ShK) family of peptides (referred to as ShKT domains).

### Excretory/Secretory-62

Excretory/secretory-62 is a phosphorylcholine (PC)-containing glycoprotein from the ES of the rodent nematode *Acanthocheilonema viteae* (124). ES-62 is known to inhibit the activation of B cells and T cells (125, 126) and has also been found to polarize antibody production through increased serum levels of IgG1 but not IgG2a (127). ES-62 affects B cells by stimulating the regulatory cytokine IL-10 and inducing a hyperresponsiveness to antigen (128). Due to its immunomodulatory potential, ES-62 was tested in an induced RA mouse model and was found to reduce disease severity and progression when administered following disease onset (129). ES-62 was also therapeutically effective in a mouse model of systemic lupus erythematosus (SLE) (84). Recently, two small synthetic molecule analogs, based on the active PC-moiety, have been shown to be effective in the mouse models of RA (82) and SLE (84).

### Neutrophil Inhibitory Factor (NIF)

Neutrophil inhibitory factor (NIF) is a glycoprotein from the ES of the canine hookworm *A. caninum* (130). NIF selectively binds the CD11b/CD18 complex, a pattern recognition receptor found on polymorphonuclear leukocytes. When activated, the complex plays an essential role in immune clearance through the facilitation of neutrophil adhesion to the endothelium, transmigration across the epithelia and phagocytosis of opsonized targets (131). Binding of NIF to CD11b/CD18 antagonizes function (132), making the molecule a potential candidate for treating acute and destructive inflammatory processes such as cerebral ischemic injury. In a phase 2 safety study on acute stroke patients, NIF was well tolerated over a wide dose range (133). This led to a study in acute ischemic stroke patients where it was hypothesized that NIF may improve neurological recovery through inhibition of neutrophil migration. However, NIF did not show improved clinical outcome, and the study was terminated (133). Since then there has been a number of animal models demonstrating the potential benefits of NIF in acute inflammatory diseases such as allergic lung inflammation (134) and diabetic retinopathy (79). Interestingly, evidence of homologous NIF proteins has been reported in other parasites including *F. hepatica* (135).

### Migration Inhibitory Factor (MIF)

Macrophage migration inhibitory factor (MIF), a human cytokine homolog, is from the ES of human-tropic nematodes (136). Paradoxically, mammalian MIF is thought to be pro-inflammatory and involved in a number of inflammatory diseases including asthma, RA, IBD, and psoriasis (65). Two secretory MIF homologs have been identified in nematodes: MIF-1 and MIF-2, possessing 40% and 27% identity with the mammalian protein, respectively (137). It has been shown that helminth-derived MIF interacts with the ubiquitously expressed antigen presentation protein CD74, suggesting a role in immunomodulation (138). Mammalian MIF has been found to influence macrophage migration, T cell activation (139), NK cell activation (140), and immunoglobulin synthesis (141), leading to the amplification of inflammatory responses. In contrast, studies on MIF-2, isolated from the nematode *Anisakis simplex*, have shown amelioration of disease in a DSS-induced colitis model (65) and an allergic airway inflammation model (142). The effect is mediated through Treg induction.

### Cystatins

Cystatins are a group of immunomodulatory proteins found in helminth ES products. Cystatins, along with stefins and kininogens, belong to a superfamily of cysteine protease inhibitors found across metazoan and plant taxa. Cysteine protease inhibitors are responsible for various biological and pathological processes including protein catabolism, antigen processing, and inflammation (143). Helminth-derived cystatins have been described in many parasite species including *Onchocerca volvulus* (144), *B. malayi* (145), *Nippostrongylus brasiliensis* (146), and *A. viteae* (143). These proteins produced by helminths have been found to target monocytes/macrophages both *in vivo* and *in vitro*, triggering the release of IL-10 that suppresses inflammatory T cells (147, 148). The cystatin from *A. viteae* was found to suppress both DSS-induced colitis and allergic lung inflammation in mice (66). In a murine model of asthma, treatment with recombinant cystatin prevented Th2 development of disease. Compared with controls, treated mice has significantly reduced eosinophil recruitment, reduced numbers of autoimmune T cells, reduced IL-4, and reduced total IgE. In a murine model of colitis, cystatin-treated mice showed significant decreases in inflammatory index and reduced epithelial damage compared to controls. The mechanism of action in both disease models was mediated by macrophages and IL-10 dependent. The immunomodulating effects of cystatins have also been examined in pig intestinal inflammation, where pigs treated with transgenic probiotic-secreting *A. vitaea* cystatin possessed a significantly reduced inflammatory score and reduced infiltration of immune cells in the colon compared with controls (148).

### Helminth Defense Molecules (HDMs)

Helminth defense molecules (HDMs) are a newly discovered family of secreted immunomodulatory proteins that share biochemical and structural characteristics with the mammalian “cathelicidin-like” host defense peptides (HDP) (149). HDPs are found in both the animal and plant kingdoms and play important roles in innate immune defense against parasites, fungi, bacteria, and viruses (150). HDMs within helminth ES are thought to minimize excessive inflammation, which helps the survival of the host and in turn survival of the parasite (151). FhHDM-1 is a HDM secreted by the trematode *F. hepatica* that adopts an α-helical structure (151). FhHDM-1 binds LPS and inhibits interaction with TLRs on macrophages. The protein has been shown to protect mice from LPS-induced inflammation and, when mixed with LPS, significantly reduces TNFα and IL-1β levels in circulation. Mechanistically, FhHDM-1 works by preventing NLRP3 inflammasome activation in macrophages through inhibiting endolysosomal acidification (152).

### P28GST

P28GST is a glutathione *S*-transferase secreted by the platyhelminth blood fluke *S. mansoni* (153). P28GST modulates mucosal immunity in mice and humans by increasing Th2 cytokine production (61). Encouragingly, immunization using a recombinant P28GST protein was as effective as helminthic therapy in reducing colitis in the TNBS model; however, a pro-Th2 adjuvant was essential for activity (61). P28GST treatment produced lower local and systemic levels of IL-5 and IL-13 and encouraged eosinophil trafficking, which was crucial for therapeutic effect. P28GST has already successfully undergone phase 1 clinical trials for safety and immunogenicity studies (NCT01512277) (154) and is currently in a phase 2 trial in CD (NCT02281916) (Table 2).

### Anti-inflammatory Protein-2 (AIP-2)

Anti-inflammatory protein-2 (AIP-2) is derived from the ES of the canine hookworm *A. caninum*. Hookworm ES products have been shown to be protective in mouse models of colitis (58, 59, 155). AIP-2 was found to be one of the most abundant proteins in the hookworm ES proteome (114), and it was recently demonstrated that intranasal delivery of recombinant AIP-2 protein could suppress airway inflammation in a mouse model of asthma and suppress antigen-specific T cell proliferation in human subjects allergic to house dust mite using *in vitro* stimulation (156). Mechanistic studies showed that AIP-2 is primarily captured by mesenteric DCs and that therapeutic effect was dependent on both DCs and Tregs. In contrast to P28GST, AIP-2 suppressed eosinophil infiltration into the lungs, the site of pathology.

### TGFβ Pathway Manipulation

TGFβ is a potent regulatory cytokine important in lymphocyte and myeloid cell differentiation and function system (157). In particular, TGFβ is a key player in the induction of immunological tolerance (158) and production can be influenced by several mechanisms of parasite infection, including host homeostasis, pathogen-triggered TGFβ production, and parasite mimicry (158). TGFβ homologs/orthologs/ligands have been characterized from several species of helminth including *A. caninum* (159), *B. malayi* (160, 161), *F. hepatica* (162), *Heligmosomoides polygyrus* (163), and the *Schistosoma* genus (164–166). In the gut, the induction of regulatory cytokines such as TGFβ is important in suppressing colitis. A study using transgenic mice with T cell-specific defects in TGFβ signaling developed spontaneous colitis (166). Here, infection with *H. polygyrus* did not prevent colitis or dampen mucosal Th1 responsiveness, indicating an essential role of T cell TGFβ signaling in regulating mucosal T cell responses.

### Prostaglandin (PG) Homologs

Prostaglandin E2 belongs to a family of autocrine and paracrine acting lipids, which in mammals are known to regulate many immune responses. Several reports have described that different helminth species including *S. mansoni* (167), *T. taeniaeformis* (168), and *B. malayi* (169, 170) produce PG homologs. A recent study identified a PGE2 homolog as a major component of *Trichuris suis* ES and suggests that secretion of this homeostatic factor contributes to protective potential in inflammatory diseases (166). PGE2 directs the immunologic balance away from Th1 responses toward a Th2 type response by modulating DC polarization (171). PGE2 can also promote resolution of inflammation and subsequent tissue repair (172) with evidence showing regeneration of epithelial crypts after DSS intestinal injury (173, 174).

### ShK

ShkT domains are relatively short peptides, 36–42 amino acids in length, containing 6 conserved cysteines and other conserved residues. ShKT domains adopt a fold with two almost perpendicular stretches of helices that are linked by three disulfide bonds that stabilize the structure (175). ShKTs have been found in both the plant and animal kingdoms suggesting ancient origins (176); however, the largest family of ShKTs are found in helminths (177). ShK from the sea anemone *S. helianthus* was one of the first immune modulating peptides discovered (178). ShK blocks the voltage-gated potassium channel Kv1.3 at low picomolar concentrations (179) by binding to a shallow vestibule at the outer entrance of the channel, which occludes entrance to the pore. Kv1.3 channels are expressed on the surface of human T cells and are vital for activation by regulating membrane potential and calcium (Ca2^+^) signaling (180, 181). Kv1.3^high^ IKCal^low^ channel phenotype is found exclusively in activated human effect memory T cells (T_EM_), whereas naïve and central memory T cells (T_CM_) remain Kv1.3^low^ upon activation. In MS, myelin-reactive T cells are predominantly T_EM_ cells, exhibiting the Kv1.3^high^ IKCal^low^ phenotype after activation with myelin antigens. Therefore, selective inhibition of autoreactive T^^EM^^ cells with disulfide rich Kv1.3 blockers could be a valuable new therapeutic lead for the treatment of MS (182). A phase 1 clinical trial was conducted to assess safety, tolerability, and pharmacokinetics of the ShK peptide in healthy volunteers (NCT02446340). Given a satisfactory safety profile, a phase Ib trial was recently conducted in psoriasis patients with results yet to be published (NCT02435342) (Table 2).

### AcK1 and BmK1

A large family of ShK-related peptides have recently been discovered in helminths, including two peptides known as AcK1 and BmK1 (177). AcK1 is a 51-residue peptide found in the ES of the hookworm *A. caninum* and the human pathogen *A. ceylanicum*. BmK1 is a *C*-terminal domain of a metalloprotease from the ES of *B. malayi* (176). Both peptides have been found to adopt helical structures that closely resemble ShK. To overcome problems in folding during *de novo* production, a truncated version of AcK1 (AcK1t) was designed lacking the first nine *N*-terminal residues, and an analog of BmK1 (BmK2) was designed based on the ShK-channel interaction surface, differing from the native peptide by five residues. Both analogs fold without difficulty, yielding a well-resolved, hydrophilic-eluting product. AcK1t and BmK2 were found to block Kv1.3 channels in the low-to-mid nanomolar range, while BmK1 was found to block the channel at low micromolar concentrations. AcK1t and BmK2 were found suppress mouse T cell proliferation *in vitro* and, in human T cells suppress mitogen stimulation. The results of these studies provide evidence that helminth peptides could potentially replace probiotic worm-based therapies to treat T_EM_-mediated autoimmune diseases such as RA, MS, T1D, and psoriasis (183–185). This would avoid complications of live worm therapy, providing a safer and more controllable therapeutic for inflammatory diseases.

## The Future of Helminth-Based Therapies

The potential for helminth-based therapies to treat autoimmune diseases have been demonstrated in animal models and clinical trials highlighted in this review. To date, the majority of clinical trials treat patients with live helminths. Justifiably, there are concerns with this method, including the associated health risks of infection with a live pathogen. However, there is the large potential to harness the specific immunomodulatory ES proteins from helminths to develop more traditional “pill”-based treatments. The synthetic production of ES-derived immunomodulators would alleviate concerns associated with live infection, and they can be produced recombinantly in high quantities at relatively low cost (186). In addition, the molecules could be directly delivered to the site of pathology for diseases such as IBD using probiotic carries that secrete the drug (187). Large-scale technologies such as genomics, proteomics, and metabolomics have increased the rate of discovery of new helminth-derived immunomodulators from the genome, and there is little doubt many more candidates will be discovered in the coming years.

## Conclusion

With the accruing global burden of autoimmune disease, helminths have become of heightened scientific interest due to their ability to activate immunoregulatory circuits and control immunity. There is strong evidence in mouse models that helminthic therapy, ES components, and helminth-derived synthetic molecules can treat and/or prevent inflammatory diseases such as IBD, T1D, MS, RA, and asthma. Thus far, human trials in celiac disease, UC, CD, MS, RA, and psoriasis have established that therapy is safe with some evidence of therapeutic effect. However, results in the first wave of human trials are not as striking as mouse disease models. Discordance in mouse/human translation is certainly not unique to this system, as is well known in other settings for a number of reasons (188). Of note, a number of the clinical studies conducted to date were not controlled, comprised small sample sizes, and/or did not use human-tropic helminths. Forthcoming trials will directly address these limitations. Going forward, the concurrent development of helminth-derived anti-inflammatory molecules provides many novel opportunities for safer and more controllable therapeutics against chronic inflammatory diseases. Indeed, inclusive efforts in characterizing and mimicking the full immunomodulating abilities of helminths are only in their infancy and much potential exists in this space.

## Author Contributions

Drafting and critical revision of the manuscript: TBS, PRG, AL, JPM, RJC, and JJM.

## Conflict of Interest Statement

The authors declare that the research was conducted in the absence of any commercial or financial relationships that could be construed as a potential conflict of interest.
